# Is Maternal Use of Paracetamol during Pregnancy Associated with Anogenital Distance in Male Newborns? The Results from the NELA Birth Cohort

**DOI:** 10.3390/ijerph18126338

**Published:** 2021-06-11

**Authors:** Fuensanta Navarro-Lafuente, Julián J. Arense-Gonzalo, Evdochia Adoamnei, María T. Prieto-Sánchez, María L. Sánchez-Ferrer, Luis García-Marcos, Eva Morales, Jaime Mendiola, Alberto M. Torres-Cantero, the NELA Study Group

**Affiliations:** 1 School of Medicine, University Campus of Espinardo, University of Murcia, 30100 Murcia, Spain; fuensanta.navarro1@um.es (F.N.-L.); evdochia.adoamnei@um.es (E.A.); mt.prieto@um.es (M.T.P.-S.); marisasanchezferrer1@gmail.com (M.L.S.-F.); lgmarcos@um.es (L.G.-M.); evamorales@um.es (E.M.); jaime.mendiola@um.es (J.M.); amtorres@um.es (A.M.T.-C.); 2 Biomedical Research Institute of Murcia (IMIB-Arrixaca), 30120 Murcia, Spain; 3 “Virgen de la Arrixaca” University Clinical Hospital, 30120 Murcia, Spain; 4 Network of Asthma and Adverse and Allergic Reactions (ARADyAL), Instituto de Salud Carlos III, 28029 Madrid, Spain; 5 Consortium for Biomedical Research in Epidemiology and Public Health (CIBER Epidemiología y Salud Pública, CIBERESP), Instituto de Salud Carlos III, 28029 Madrid, Spain

**Keywords:** paracetamol, acetaminophen, anogenital distance, AGD, endocrine disruptors, pregnancy

## Abstract

Paracetamol is the one of the most commonly used medications during pregnancy. However, its potential antiandrogenic effect has been suggested. The objective of this study was to evaluate associations between maternal paracetamol use during pregnancy and anogenital distance (AGD) in male newborns from a Spanish birth cohort. The study included two hundred and seventy-seven mother-male child pairs with self-reported paracetamol use and frequency during each trimester of pregnancy. AGD measurements were taken employing standardized methods. The associations between maternal paracetamol use and AGD measures were evaluated using linear regression models, adjusting for potential confounders and covariates. Overall, 61.7% of pregnant women consumed paracetamol at any time of pregnancy with an average of 9.43 (SD = 15.33) days throughout pregnancy. No associations between the maternal use of paracetamol or its frequency and AGD measures among different trimesters or during the whole pregnancy were found in the adjusted final models. A non-differential misclassification error may have occurred—the recall of paracetamol intake independent of AGD measurements—introducing bias towards the null hypothesis. Nevertheless, the current evidence suggests that paracetamol might have a potential antiandrogenic effect especially in the early stages of fetal development. Thus, it would be highly recommendable to pursue further studies to elucidate the potential effects of paracetamol in human perinatal health and its use among pregnant women.

## 1. Introduction

Paracetamol (acetaminophen) is the most widely used international analgesic and antipyretic and it is recommended as a first-line therapy in the treatment of mild-to-moderate pain by the World Health Organization (WHO) [1]. Furthermore, paracetamol is one of the most commonly used medications during pregnancy [2,3], being classified as a low-risk for gestation [4,5]. However, recent investigations have suggested that paracetamol exposure during pregnancy may affect several child health outcomes including alterations to the endocrine system and hormonal regulation [2]. The mechanisms underlying these findings may be related to the potential antiandrogenic effect of paracetamol [6,7,8]. Experimental trials in rodents have demonstrated that intra-uterine exposure to paracetamol reduces plasma testosterone levels through a reduction of its secretion by Leydig cells in fetal testicles [8,9,10]. Nonetheless, as the European Medicines Agency indicated in 2019, epidemiological studies in children whose mothers consumed paracetamol during pregnancy showed unclear results [11]. Moreover, international and national health organizations have stated that the use of paracetamol in therapeutic doses and for the shortest possible time does not produce adverse effects on pregnant women, the fetus or newborns [11,12].

Anogenital distance (AGD) is a biomarker of the intra-uterine hormonal environment. It has been demonstrated that AGD depends largely on androgen levels to which the fetus was exposed during prenatal life [13,14,15]. Particularly, this relationship is of great interest during the first trimester of pregnancy, known as the masculinization programming window (MPW) [16,17]. In this period, a sufficient exposure to androgens is necessary to ensure the subsequent adequate development and differentiation of the male reproductive tract and function [18].

Recent studies in large birth cohorts have found an association between maternal paracetamol consumption during pregnancy and a shorter AGD in their male offspring [18,19]. In this line, the relationship between AGD and maternal paracetamol consumption might be due to the potential antiandrogenic action of paracetamol. This action could disrupt the masculinization process, resulting in a shorter AGD in male children [6,9,10]. Moreover, as Sharpe (2020) indicated recently, maternal lifestyle and diet during pregnancy including the use of painkillers may be more important than other environmental chemical exposures in the origin of several reproductive pathologies in humans.

Overall, the current knowledge of the potential effect of paracetamol on prenatal male reproductive system development is still scarce and inconclusive. Therefore, it is relevant to explore and evaluate these potential associations in other birth cohorts. Thus, our objective was to assess whether maternal paracetamol use during pregnancy was associated with AGD measures in their male offspring in the Spanish birth cohort ‘Nutrition in Early Life and Asthma’ (NELA).

## 2. Material and Methods

### 2.1. Study Population

The data came from participants embedded in the Nutrition in Early Life and Asthma (NELA) study (www.nela.imib.es), a prospective population-based birth cohort set up in Murcia, a south-eastern Mediterranean region of Spain. The main objective of NELA was to unravel the developmental origins and mechanisms of asthma and allergies.

Pregnant women who fulfilled the inclusion criteria were invited to participate in the study at the time of the control ultrasound at 20 weeks of gestation at the Maternal-Fetal Unit of the Virgen de la Arrixaca University Hospital over a 36 month period from March 2015 to April 2018. The inclusion criteria were: women from Health Area I and certain districts of Health Areas VI and VII of the Region of Murcia; planning to live in the area of study for at least two years; the intention to give birth at the reference hospital; Spanish Caucasian origin; 18–45 years of age; singleton pregnancy; spontaneous conception and normal echography at 20 weeks of gestation (no major fetal malformations). The exclusion criteria included: an existing maternal chronic disease (pre-gestational diabetes mellitus or other major endocrine disorders, pre-gestational hypertension, autoimmune disease or cancer) and verbal communication problems. Among the 1350 women invited to participate, 738 (54%) were finally enrolled in the study. Of these women, one hundred and forty-nine were excluded because the newborns did not have the AGD measured and eight mothers did not report information about their paracetamol use. Finally, 277 of the remaining 561 pairs had male children and completed information; hence, they were enrolled in the current analysis.

For this study, the sample size was calculated following clinical data from previous publications [18,19]. It was considered that it would be appropriate to detect a difference of at least 2 mm (with a standard deviation of about 6 mm) in AGD measurements between both groups (maternal use of paracetamol during pregnancy vs. no maternal use of paracetamol during pregnancy). For an alpha error of 0.05 and 80% statistical power to detect the differences, a minimum of 274 mother-child pairs was required.

This study was approved by the Ethics and Clinical Research Committee (CEIC) of the Virgen de la Arrixaca Clinical University Hospital and the Research Committee of University of Murcia in accordance with the guidelines of The Declaration of Helsinki. Written informed consent was obtained from all participants.

### 2.2. Data Collection: Sociodemographic and Physical Examination

The following information was collected through questionnaires applied by the trained personnel of the study to mothers: maternal age; marital status; maternal educational level (basic education, incomplete secondary, complete secondary and university); maternal social class (defined as maternal occupation during pregnancy based on the highest social class by using a widely used Spanish adaptation of the international ISCOSS coding system: I/II, managers/technicians/graduated; III, skilled; IV–V, semi-skilled/unskilled and unemployed) [20]); obstetric history (previous gestations, deliveries and abortions/miscarriages), maternal intake of alcohol and smoking status during pregnancy (yes/no), maternal weight before pregnancy (kg) and weight gain throughout the pregnancy (kg). The height (m) of pregnant women was measured by qualified technical personnel of the study. The maternal BMI before pregnancy was calculated (weight before pregnancy (kg)/height (m)^2^) and pregnant women were classified within the following ranges: normal weight (BMI < 25), overweight (BMI = 25–29.99) and obesity (BMI > 30).

The mothers had three follow-up visits; one in the second trimester of pregnancy (between 20 and 24 weeks), one in the third trimester (between 32 and 36 weeks) and one at delivery. Further information was obtained from the clinical registrations at the time of delivery: gestational age at delivery (weeks), newborn gender, birth weight (g) and height (cm). The medical diagnosis of pregnancy complications such as the risk of miscarriage, hypertension or preeclampsia and gestational diabetes mellitus was also registered.

Women reported the use of medications and other supplements during pregnancy at the clinic visit of the 32nd week. More specifically, paracetamol consumption (yes/no) data were available for each trimester of gestation as well as the days of treatment.

The AGD measurements were carried out during the anthropometric examination of the newborns 24–48 h after birth. For each male newborn, the AGD was measured in two ways: AGD_AP_ (anus-penis) and AGD_As_ (anus-scrotum) using a digital stainless steel caliber (VWR International, LLC, West Chester, PA, USA). The measurement methods have been described elsewhere [21,22]. To improve accuracy and assess intra-examiner reproducibility, two examiners performed each measurement three times and the mean of these three was used as an estimate. The intra-class correlation coefficient (ICC) between the two examiners and the coefficient of variations (CVw) within each one in a subsample (approximately 10% of the total population) were calculated (Supplementary Table S1).

### 2.3. Statistical Analysis

A descriptive analysis of the study sample was carried out for both mothers and newborns. The continuous variables were summarized by the arithmetic mean and standard deviation (SD) and the categorical variables as a number and percentage (%).

The differences of the main characteristics of women and the AGDs of male children according to the use of paracetamol (yes/no) at any time during pregnancy and by trimester were assessed. The assessment for normality was performed using a Kolmogorov–Smirnov test. A chi-squared test was used for the categorical variables and a Student’s *t*-test or Mann–Whitney U-test was used for the quantitative variables. Pearson or Spearman coefficients were employed to evaluate the potential correlations between variables.

Linear regression models were used to evaluate the associations between maternal paracetamol use at any time during pregnancy and in each gestational trimester and AGD measures in male newborns. The AGD measurements were transformed into Z-scores and parameter estimates were reported with beta and 95% confidence intervals (CI). The AGD Z-scores were calculated based on the 277 baby boys. Several potential covariates and confounders were evaluated and, when an inclusion of a potential covariate resulted in a change in the β-coefficient of <10%, the variable was not retained in the final models. These variables included factors previously related to AGD in this or other studies regardless of whether they had been previously described as predictors of male reproductive health in early stages. Lastly, previous pre-term deliveries and gestational age were the variables retained in the final models.

We also evaluated the associations between the frequency of maternal use of paracetamol (days) and AGD measures in the first trimester and at any time during pregnancy using an analysis of the covariance (ANCOVA) taking into account the same covariates mentioned earlier. The days of consumption were categorized in intervals: Never; 1–3 days; 4–7 days; 1–2 weeks and more than 2 weeks. This categorization was based on previous animal and human studies in which the effects of exposure to paracetamol during pregnancy were evaluated according to the exposure time or doses used [8,9]. For the statistical analyses, the software package IBM SPSS 20.0 was employed (IBM Corporation, Armonk, NY, USA), assuming a 0.05 significance level for all tests.

## 3. Results

The women had a mean age of 32.8 (SD = 4.5) years and there were significant differences regarding the number of previous gestations, deliveries and pre-term deliveries, which were higher in women who reported a consumption of paracetamol compared with women who did not (*p*-value < 0.05). No differences in other sociodemographic and gynecologic characteristics were found between both groups (Table 1).

The percentage of pregnant women who reported the use of paracetamol at any time of pregnancy was 61.7%. Among different trimesters of pregnancy, 102 (36.8%) women consumed paracetamol in the first trimester, 112 (40.4%) in the second and 96 (54.5%) in the third (Table 2).

In the bivariate analyses, there were no differences in male AGD measurements when any trimester of paracetamol use was evaluated (Table 2). However, when AGDs were analyzed according to the consumption of paracetamol by trimesters, significant differences in AGD_AP_ in the first trimester of pregnancy were found (46.3 ± 5.2 mm (maternal use of paracetamol) vs. 44.9 ± 5.5 mm (no maternal use of paracetamol); *p*-value = 0.04). This association remained significant in crude linear regression models (Table 3). However, no significant associations between the maternal use of paracetamol at any time during pregnancy or by trimesters and AGD measurements were found in the final adjusted models (Table 3). Moreover, a model with AGD measures without a Z-score and taking into account birth weight and gestational age was fitted. In this case, a significant positive relationship between paracetamol use and AGD_AP_ in the first trimester of pregnancy was shown (β = 1.23, 95%CI: 0.06; 2.40, *p*-value = 0.04). No other changes were observed regarding any other results.

The descriptive values of the days of maternal paracetamol use during pregnancy can be found in Supplementary Table S2. Pregnant women had an average use of paracetamol of 9.43 (SD = 15.33) days when the consumption was considered at any time of the pregnancy. Furthermore, the average consumption in the first trimester was 5.48 days (SD = 6.55), 6.15 (SD = 9.58) in the second and 4.1 (SD = 4.63) days in the third. Overall, no differences in the AGD measurements were found between the intervals of maternal days of paracetamol consumption either at any time of pregnancy or in the first trimester (Table 4) in the final adjusted models.

## 4. Discussion

In our study, no differences in male AGD measures were found between male offspring whose mothers consumed paracetamol during pregnancy and those who did not. Additionally, the frequency of maternal paracetamol use was not associated with any AGD measurements. To the best of our knowledge, this is the first study evaluating this matter in a birth cohort from southern Europe.

Only two human observational studies have attempted to evaluate the associations between maternal paracetamol use during pregnancy and AGD measurements in their male children. In agreement with our results, the study on the Odense Child Cohort showed that exposure to paracetamol was not related to changes in the AGD measurements in male offspring [19]. However, the combined maternal use of paracetamol and non-steroidal anti-inflammatory drugs (NSAIDs) (*n* = 20) during the first half of pregnancy (<28 weeks) was significantly associated with a shorter AGD_AS_ at three months of age [19].

In contrast, another birth cohort study did show differences in male AGD measures between children who were prenatally exposed to paracetamol without a combination with other analgesics and those who were not, especially in the early stages [18]. In this case, the Cambridge Baby Growth Study reported an association between paracetamol exposure and a shorter male AGD between birth and 24 months of age (by SD = 0.27, *p*-value = 0.014) but only when the use was during the period from 8 to 14 weeks of gestation, concurrent with the human MPW. No differences in AGD measures were observed if the maternal consumption of paracetamol occurred either during the first weeks of gestation (<8 weeks) or during the rest of pregnancy (>14 weeks) [18].

A few methodological differences should be pointed out regarding the two comparable published articles [18,19]. For example, AGD was measured as a single distance from the center of the anus to the scrotum at different times from birth (0, 3, 12, 18 and 24 months of age) in the Cambridge Baby Growth Study cohort [18] and the associations between AGD measures and paracetamol exposure were reported as differences between AGDs measured at different times. However, the genital measurements of male babies in the Odense Child Cohort were more similar to those used in our cohort and based on a standardized methodology but three months after birth [19]. In our case, both AGD measures were taken at birth. Overall, paracetamol exposure during pregnancy was collected through retrospective questionnaires in these two previous studies [18,19] as well as in ours in which mothers self-reported their use of paracetamol and other medications. However, in the Cambridge cohort, only one single time was set to questions about maternal medication during pregnancy before birth. Therefore, a non-differential misclassification error may be considerably higher than in other studies, including ours, in which women were asked to complete information about medication use two or three times during pregnancy. Overall, there is no unified or standardized collection method to capture the use of these medications, which hampers the comparison and reproducibility of the investigations.

We performed an analysis taking AGD measures without a Z-score and accounting for birth weight and gestational age. A significant positive relationship between paracetamol use and AGD_AP_ in the first trimester of pregnancy was observed. In this regard, the statistical significance was near the known threshold (*p*-value = 0.04) and, taking into account the potential non-differential misclassification error, a cautious interpretation of this finding might be needed. Moreover, a potential chance finding cannot be completely ruled out either and the explanation of the result might be uncertain. Lastly, the two previous studies investigating the same matter used AGD Z-scores values; thus, taking the same approach would allow us to compare more appropriately our results with others as we did throughout the manuscript.

Despite our results, several experimental studies in rats have reported associations between individual exposure to paracetamol or other mild analgesics and the inhibition of testosterone secretion by Leydig cells [9,10,23]. Furthermore, human observational studies have shown a direct correlation between testosterone-dependent pathologies in male offspring and the maternal use of paracetamol during pregnancy (reviewed by Kilcoyne and Mitchel, 2017) [24], supporting the hypothesis about the potential antiandrogenic effects of this drug [16]. For example, in the Danish cohort, the use of paracetamol was dose-dependently associated with the occurrence of congenital cryptorchidism. Moreover, in this population, the risk of presenting that condition at birth was also increased with the maternal combined use of two or more analgesics during the second trimester [9] and with an exposure for more than four weeks [25]. In this context, the current evidence suggests that the timing and duration of prenatal paracetamol exposition might have important implications [23]. However, to our knowledge, our study is the first one to evaluate the potential relationship between the frequency of maternal paracetamol use during pregnancy and AGD measurements in humans.

Regarding potential underlying mechanisms, in animal experiments with pregnant rats consuming paracetamol orally, decreased testosterone levels in male offspring have been shown as down-regulated steroidogenic enzymes (Cyp11a1 and Cyp17a1), resulting in a shorter AGD as well [6,8]. The administration of this drug in therapeutic doses directly reduced the plasma levels of testosterone (45% reduction; *p*-value = 0.02) and the weight of the seminal vesicle (18% reduction; *p*-value = 0.005), which is an androgen exposure biomarker [8]. Moreover, an in vitro study with human fetal testes showed that paracetamol decreased insulin-like factor 3 levels and inhibited prostaglandin (PGE_2_) levels, which are important mediators of the endocrine function [26]. Additionally, these findings were later supported by Hurtado-González and co-workers in 2018, whose investigation concluded that PGE_2_ could mediate the effects of paracetamol on human germ cells in fetal testis and ovaries [7].

Our study is not without limitations. First, we used a questionnaire to collect information about the use of medications during pregnancy. Pregnant women self-reported their paracetamol use, which could be a source of exposure measurement error. However, the mothers did not know the purpose of this questionnaire at the time of compilation therefore it was not influenced by any previous considerations. A non-differential misclassification error due to the recall of paracetamol intake may have biased our results towards the null hypothesis, influencing the potential associations with the AGD measurements. This bias may become even more pronounced when assessing the days of usage and potential dose. Therefore, the potential effect of this bias in the observed null associations cannot be ruled out at all. Second, the relatively high prevalence of paracetamol use in our study population (36.8–61.7%) was similar to other observational studies; for example, 58% of pregnant women from the MotherToBaby study (USA) [27] or 47% of women included in the Danish National Birth Cohort [25]. However, our study did not consider other relevant variables regarding paracetamol use such as the specific doses, medical prescription or combination with other mild analgesics. Third, bias due to errors in the AGD measurements might have occurred. However, we obtained acceptable (fair) intra- and inter-examiner reliability and the methodology has been widely used before. Finally, the relatively small sample size of our sample might decrease the probability of finding significant differences between maternal paracetamol use and the AGD in male offspring. Nonetheless, our study was appropriately powered to detect statistically significant differences in AGD measures reported in previous published articles on the matter.

## 5. Conclusions

Our study did not find associations between maternal use and frequency of use of paracetamol during pregnancy and the AGD measurements in their male offspring. However, a non-differential misclassification error may have occurred—the recall of paracetamol intake independent of the AGD measurements—introducing bias towards the null hypothesis. Despite our findings, the available evidence suggests a potential antiandrogenic effect of paracetamol. However, our results were not consistent with that hypothesis. Therefore, future studies are needed to evaluate if its effect as an endocrine disruptor in early stages of gestation (which animal studies have shown) can be consistently extrapolated to humans. If it is replicated, a biomonitoring of serum or urine paracetamol levels and a prospective control of frequency and dose of administration in combination with other medications (by weekly questionnaires or interviews) should be considered in order to avoid measurement bias.

## Figures and Tables

**Table 1:** Characteristics of participants in the current study (mean and standard deviation (SD) or *n* (%)). *p*-value ≤ 0.05 is considered statistically significant.

**Characteristics of Pregnant Women**	**All Women** **(*n = 277*)**	**Use of Paracetamol at Any Time during Pregnancy**		
		**Yes (*n = 171*)**	**No (*n = 106*)**	***p*-Value ^a^**
Age (years)	32.8 (4.5)	32.7 (4.3)	33.0 (4.6)	0.6
Current marital status: Married Single Divorced	271 (97.8%) 5 (1.8%) 1 (0.4%)	168 (98.2%) 2 (1.2%) 1 (0.6%)	103 (97.2%) 3 (2.8%) 0 (0%)	0.4
Educational level: Basic education Incomplete secondary education Secondary education University education	16 (5.8%) 35 (12.6%) 73 (26.4%) 153 (55.2%)	8 (4.7%) 25 (14.6%) 49 (28.7%) 89 (52.0%)	8 (7.5%) 210 (9.4%) 24 (22.6%) 64 (60.4%)	0.3
Social class: I–II managers/technicians/graduated III skilled IV–V semi-skilled/unskilled Unemployed	104 (37.5%) 80 (28.9%) 43 (15.5%) 50 (18.1%)	65 (38.0%) 44 (25.7%) 29 (17.0%) 33 (19.3%)	39 (36.8%) 36 (34.0%) 14 (13.2%) 17 (16.0%)	0.5
Maternal height before pregnancy (cm)	164.4 (5.9)	164.4 (5.8)	164.5 (6.2)	0.9
Maternal weight before pregnancy (kg)	64.8 (12.9)	65.5 (14.1)	63.3 (11.1)	0.2
Maternal BMI before pregnancy (kg/m^2^)	23.9 (4.3)	24.2 (4.7)	23.4 (3.6)	0.1
Maternal BMI before pregnancy: Normal weight Overweight Obese	208 (72.2%) 57 (19.8%) 23 (8.0%)	120 (70.6%) 32 (18.8%) 18 (10.6%)	81 (76.4%) 21 (19.8%) 4 (3.8%)	0.1
Maternal weight at 32nd week (kg)	77.5 (12.6)	77.9 (13.8)	76.4 (10.5)	0.4
Maternal weight gain (kg)	12.3 (5.4)	12.4 (5.6)	12.3 (5.0)	0.9
Previous gestations (*n*)	1.0 (1.2)	1.1 (1.2)	0.8 (1.0)	0.02
Previous miscarriages (*n*)	0.4 (0.8)	0.4 (0.8)	0.3 (0.7)	0.6
Previous deliveries (*n*)	0.6 (0.7)	0.7 (0.8)	0.4 (0.6)	0.003
Previous pre-term deliveries (<37 weeks) (*n*)	0.05 (0.2)	0.1 (0.3)	0.02 (0.1)	0.04
Risk of miscarriage in the current pregnancy: No Yes	264 (95.3%) 13 (4.7%)	164 (95.9%) 7 (4.1%)	100 (94.3%) 6 (5.7%)	0.5
Gestational Diabetes Mellitus: No Yes	251 (90.6%) 26 (9.4%)	155 (90.6%) 16 (9.4%)	96 (90.6%) 10 (9.4%)	0.9
Gestational Hypertension: No Yes	269 (97.1%) 8 (2.9%)	165 (96.5%) 6 (3.5%)	104 (98.1%) 2 (1.9%)	0.4
Pre-eclampsia: No Yes	255 (99.2%) 2 (0.8%)	157 (61.6%) 1 (0.6%)	98 (99.0%) 1 (1.0%)	0.7
Maternal alcohol intake: No Yes	257 (92.8%) 20 (7.2%)	158 (92.4%) 13 (7.6%)	99 (93.4%) 7 (6.6%)	0.7
Smoking status: No Yes	239 (86.3%) 38 (13.7%)	148 (86.5%) 23 (13.5%)	91 (85.8%) 15 (14.2%)	0.9
**Characteristics of Newborns**	**Male Newborns** ***(n = 277)***	**Use of Paracetamol at Any Time during Pregnancy**		
		**Yes *(n = 171)***	**No *(n = 106)***	***p*-value ^a^**
Gestational age at delivery(weeks)	39.6 (1.4)	39.6 (1.4)	39.5 (1.4)	0.5
Newborn height (cm)	51.0 (2.2)	51.1 (2.1)	50.8 (2.3)	0.4
Newborn weight (gr)	3288.4 (477.0)	3295.8 (470.6)	3268.3 (458.8)	0.6

^a^ Student’s *t*-test, Mann–Whitney U-test or chi-squared test.

**Table 2:** Anogenital distance measurements of males (N = 277) at birth cohort NELA according to the maternal use of paracetamol during pregnancy. *p*-value ≤ 0.05 is considered statistically significant.

AGD Measures of Male Newborns (*n = 277*)		Maternal Use of Paracetamol											
		Any Trimester			First Trimester			Second Trimester			Third Trimester		
		Yes	No	*p*-Value ^a^	Yes	No	*p*-Value ^a^	Yes	No	*p*-Value ^a^	Yes	No	*p*-Value ^a^
	Mean Value (SD)	*n = 171*	*n = 106*		*n = 102*	*n = 175*		*n = 112*	*n = 165*		*n = 96*	*n = 80*	
AGD_AS_ (mm)	23.3 (4.9)	23.4 (4.7)	23.3 (5.3)	0.9	23.7 (4.5)	23.1 (5.1)	0.4	23.5 (4.8)	23.2 (5.0)	0.6	23.9 (4.9)	23.0 (4.9)	0.2
AGD_AP_ (mm)	45.4 (5.4)	45.5 (5.5)	45.3 (5.2)	0.7	46.3 (5.2)	44.9 (5.5)	0.04	45.1 (5.3)	45.6 (5.5)	0.5	45.6 (5.9)	45.3 (5.2)	0.9

AGD_AS_: anogenital distance from the anus to the posterior base of the scrotum; AGD_AP_: anogenital distance from the anus to the cephalad insertion of the penis. ^a^ Student’s *t*-test or Mann–Whitney U-test. AGDs were transformed into Z-scores.

**Table 3:** Unadjusted and adjusted linear regression models of male anogenital distance according to the maternal use of paracetamol in pregnancy. *p*-value ≤ 0.05 is considered statistically significant.

Measures of Male Newborns (*n = 277*)		Maternal Use of Paracetamol											
		Any Trimester			First Trimester			Second Trimester			Third Trimester		
	Mean Value (SD) (mm)	***n* (Exposed, Not Exposed)**	**Parameter Estimate ^a^**	***p*-Value**	***n* (Exposed, Not Exposed)**	**Parameter Estimate ^a^**	***p*-Value**	***n* (Exposed, Not Exposed)**	**Parameter Estimate ^a^**	***p*-Value**	***n* (Exposed, Not Exposed)**	**Parameter Estimate ^a^**	***p*-Value**
		**CRUDE MODEL ^b^**											
AGD_AS_	23.3 (4.9)	171, 106	0.1 (*−*0.23, 0.25)	0.94	102, 175	0.12 (*−*0.12, 0.36)	0.34	112, 165	0.08 (*−*0.16, 0.32)	0.51	96, 181	0.19 (*−*0.05, 0.44)	0.13
AGD_AP_	45.4 (5.4)	171, 106	0.04 (*−*0.21, 0.28)	0.76	102, 175	0.26 (0.01, 0.5)	0.04	112, 165	−0.1 (*−*0.34, 0.14)	0.42	96, 181	0.05 (*−*0.2, 0.3)	0.68
		**ADJUSTED MODEL ^c^**											
AGD_AS_	23.3 (4.9)	171, 106	−0.003 (*−*0.23, 0.24)	0.98	102, 175	0.09 (*−*0.15, 0.33)	0.46	112, 165	0.09 (*−*0.14, 0.33)	0.42	96, 181	0.2 (*−*0.04, 0.44)	0.1
AGD_AP_	45.4 (5.4)	171, 106	0.03 (*−*0.2, 0.26)	0.81	102, 175	0.22 (*−*0.01, 0.45)	0.07	112, 165	−0.08 (*−*0.31, 0.15)	0.49	96, 181	0.06 (*−*0.17, 0.3)	0.6

AGD_AS_: anogenital distance from the anus to the posterior base of the scrotum; AGD_AP_: anogenital distance from the anus to the cephalad insertion of the penis. **^a^** Parameter estimate = beta (95%CI). **^b^** Unadjusted lineal regression models. AGDs were transformed into Z-scores. **^c^** Lineal regression models adjusted by previous pre-term deliveries and gestational age. AGDs were transformed into Z-scores.

**Table 4:** Male anogenital distance according to the days of maternal use of paracetamol at any time of the pregnancy and during the first trimester (Mean (SD)). *p*-value ≤ 0.05 is considered statistically significant.

**Male Newborns (*n = 277*)**	**Days of Maternal Use of Paracetamol at Any Time of Pregnancy**						
	**Never (*n = 105*)**	**1–3 Days (*n = 70*)**	**4–7 Days (*n = 46*)**	**1–2 Weeks (*n = 28*)**	**More Than 2 Weeks (*n = 26*)**	***p*-Value ^a^**	***p*-Value ^b^**
AGD_AS_ (mm)	23.3 (5.3)	23.0 (4.7)	23.2 (4.5)	23.9 (4.3)	23.7 (5.3)	0.93	0.86
AGD_AP_ (mm)	45.3 (5.3)	45.3 (5.8)	45.1 (5.3)	46.1 (5.1)	45.9 (5.6)	0.92	0.79
**Days of Maternal Use of Paracetamol in The First Trimester**							
	**Never (*n = 176*)**	**1–3 Days (*n = 58*)**	**4–7 Days (*n = 24*)**	**1–2 Weeks (*n = 8*)**	**More than 2 Weeks (*n = 11*)**	***p*-Value ^a^**	***p*-Value ^b^**
AGD_AS_ (mm)	23.1 (5.1)	23.5 (4.5)	24.1 (3.8)	23.4 (5.9)	23.6 (5.4)	0.89	0.91
AGD_AP_ (mm)	44.9 (5.5)	46.7 (5.1)	44.9 (5.2)	48.0 (5.6)	46.2 (5.1)	0.12	0.13

AGD_AS_: anogenital distance from the anus to the posterior base of the scrotum; AGD_AP_: anogenital distance from the anus to the cephalad insertion of the penis. ^a^ANOVA test. AGDs were transformed into Z-scores. ^b^ANCOVA test. AGDs were transformed into Z-scores. Models adjusted by previous pre-term deliveries and gestational age.

## Data Availability

The data that support the findings of this study are restricted for research use only. The data are not publicly available. Data are available from the authors upon reasonable request and with permission from the Executive Committee of the NELA project.

## Supplementary Materials

The following are available online at https://www.mdpi.com/article/10.3390/ijerph18126338/s1; Table S1 Coefficients of variation within (CVw) examiner and intra-class correlation coefficients (ICC) between two examiner for AGD measurements, Table S2 Descriptive values of days of maternal paracetamol use during pregnancy.
