# Supplementary material for: Is Maternal Use of Paracetamol during Pregnancy Associated with Anogenital Distance in Male Newborns? The Results from the NELA Birth Cohort
Source: Int J Environ Res Public Health. 2021 Jun 11;18(12):6338. doi: 10.3390/ijerph18126338 (PMC8296170; doi:10.3390/ijerph18126338)
Supplement: Supplementary file 1 [file ijerph-18-06338-s001.zip › ijerph-1236147-supplementary.pdf]

Supplementary Table S1. Coefficients of variations within (CVw) examiner and intra-class correlation coefficients (ICC) between two examiners for AGD measurements.

Measure of male newborns (N= 35)	Examiner	CVw (%) [a]	ICC
AGD_{AS}	1	3.6	0.50
	2	3.5	
AGD_{AP}	1	2.0	0.60
	2	1.8	

AGD_{AS}: Anogenital Distance from the anus to the posterior base of the scrotum; AGD $_{AP}$: Anogenital Distance from the anus to the cephalad insertion of the penis.
[a] Three measurements each time.

Supplementary Table S2. Descriptive values of days of maternal paracetamol use during pregnancy.

Period during pregnancy	N (%)	Days of maternal use of paracetamol during pregnancy	
		Mean (SD)	Median (P5-P95)
First trimester	101 (34.9%)	5.48 (6.55)	3.0 (1-22.8)
Second trimester	110 (38.0%)	6.15 (9.58)	3.0 (1-24.0)
Third trimester	93 (32.2%)	4.01 (4.63)	3.0 (1-15.0)
Any time during pregnancy	170 (58.5%)	9.43 (15.33)	5.0 (1-40.0)

P5-P95: Percentile 5-Percentile 95. SD: Standard deviation.
